# 225Ac‐labeled CD33‐targeting antibody reverses resistance to Bcl‐2 inhibitor venetoclax in acute myeloid leukemia models

**DOI:** 10.1002/cam4.3665

**Published:** 2020-12-21

**Authors:** Ravendra Garg, Kevin J. H. Allen, Wojciech Dawicki, Eileen M. Geoghegan, Dale L. Ludwig, Ekaterina Dadachova

**Affiliations:** ^1^ University of Saskatchewan Saskatoon Canada; ^2^ Actinium Pharmaceuticals Inc New York USA

**Keywords:** 225Ac‐lintuzumab, acute myeloid leukemia, Bcl‐2, radioimmunotherapy, venetoclax

## Abstract

**Purpose:**

Despite the availability of new drugs, many patients with acute myeloid leukemia (AML) do not achieve remission and outcomes remain poor. Venetoclax is a promising new therapy approved for use in combination with a hypomethylating agent or with low‐dose cytarabine for the treatment of newly diagnosed older AML patients or those ineligible for intensive chemotherapy. ^225^Actinium‐lintuzumab (^225^Ac‐lintuzumab) is a clinical stage radioimmunotherapy targeting CD33 that has shown evidence of single‐agent activity in relapsed/refractory AML. Increased expression of MCL‐1 is a mediator of resistance to venetoclax in cancer.

**Experimental design:**

Here we investigated the potential for ^225^Ac‐lintuzumab‐directed DNA damage to suppress MCL‐1 levels as a possible mechanism of reversing resistance to venetoclax in two preclinical in vivo models of AML.

**Results:**

We demonstrated that ^225^Ac‐lintuzumab in combination with venetoclax induced a synergistic increase in tumor cell killing compared to treatment with either drug alone in venetoclax‐resistant AML cell lines through both an induction of double‐stranded DNA breaks (DSBs) and depletion of MCL‐1 protein levels. Further, this combination led to significant tumor growth control and prolonged survival benefit in venetoclax‐resistant in vivo AML models.

**Conclusions:**

There results suggest that the combination of ^225^Ac‐lintuzumab with venetoclax is a promising therapeutic strategy for the treatment of patients with venetoclax‐resistant AML.

Clinical trial of this combination therapy (NCT03867682) is currently ongoing.

## Graphical abstract

Long‐term survival of adult patients with acute myeloid leukemia (AML) remains unsatisfactory.We describe the results of a mechanistic and efficacy study combining venetoclax (ABT‐199), a recently approved BCL2 inhibitor, and the clinical stage CD33‐targeting antibody radiolabeled with ^225^Actinium (^225^Ac‐lintuzumab) in two venetoclax‐resistant AML models in vitro and in vivo. The study demonstrated dramatic synergy of these two drugs both in vitro and in vivo due to the decrease in BCL‐2 and MCL‐1 levels and induction of double DNA strand breaks. Since both venetoclax and ^225^Ac‐lintuzumab are already used in patients, we anticipate that rapid translation of this combination approach into the clinic will benefit patients with AML.

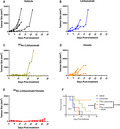

## 1 INTRODUCTION

Although standard induction therapy with cytarabine and anthracycline produces complete remissions (CR) in 60% to 80% of younger adults with acute myeloid leukemia (AML), long‐term survival is seen in only 25% to 50% of patients.^1^ Following relapse, salvage chemotherapy produces remissions in only 20% to 25% of patients. Further, while allogeneic hematopoietic cell transplantation (HCT) can result in long‐term survival in approximately 30% to 40% of patients with relapsed AML, many patients are not suitable candidates for transplant due to age, comorbidities, or lack of a matched donor.^1^ The prognosis for older patients is even worse, with a 5‐year median survival rate of 5% for patients older than the age of 65.^2^ Within the last few years, following decades of limited advancement in the treatment of AML, several new targeted therapies have been approved in the US.^3^, ^4^ Notably, the BCL‐2 inhibitor venetoclax (ABT‐199) was approved for use in combination with a hypomethylating agent (HMA) or with low‐dose cytarabine (LDAC) for the treatment of newly diagnosed patients with AML who are age 75 years or older or are ineligible for intensive chemotherapy.^5^, ^6^ Approval was based on the M14‐358 and M14‐387 phase Ib/II trials. In M14‐358, the combination of venetoclax with azacitidine led to a complete remission (CR) rate of 37% and a CR with partial hematological recovery (CRh) rate of 24%.^5^ For the combination of venetoclax and decitabine, the rates were 54% and 7.7%, respectively. In the M14‐387 trial, venetoclax in combination with low‐dose cytarabine (LDAC) led to 21% CR and CRh rates.^6^ For patients receiving all dosages of venetoclax in these studies, median overall survival was 17.5 months.^5^ Despite the significant benefit to patients in this population, not all patients respond to initial therapy with venetoclax and most patients will eventually progress. Preclinical studies have investigated mechanisms of resistance, which are supported by clinical results in patients with progressive disease. Importantly, venetoclax as a B cell lymphoma 2 (BCL‐2) selective inhibitor does not inhibit other BCL family members such as myeloid cell leukemia 1 (MCL‐1) or B lymphoma extra‐large (BCL‐XL). Mechanistically, overexpression of these other antiapoptotic BCL‐2 family members, in particular MCL‐1, or their upregulation in response to venetoclax has been shown to mediate resistance to venetoclax in leukemia, lymphoma, and multiple myeloma.^7^, ^8^, ^9^, ^10^, ^11^ Further, MCL‐1 has been shown to be upregulated in AML patients at relapse following induction chemotherapy.^12^ Strategies to reduce MCL‐1 levels may therefore dramatically prolong the response to venetoclax and re‐sensitize resistant tumors to venetoclax therapy. MCL‐1 protein has a very short half‐life of less than 1 hour and is therefore sensitive to changes in RNA or protein synthesis.^13^ To that end, genotoxic stress as a result of DNA damage, for example, by UV or ionizing radiation, or chemo‐induced, can effect a reduction in MCL‐1 levels via inhibition of protein synthesis.^14^, ^15^ In turn, the combination with chemotherapy or ionizing radiation can increase sensitivity to BCL‐2 inhibitors in preclinical tumor cell lines and patient samples by a reduction in MCL‐1 levels.^7^, ^14^, ^16^ ^225^Ac‐lintuzumab is a clinical stage radioimmunotherapy targeting CD33 that has shown evidence of single‐agent activity in relapsed/refractory AML^17^, ^18^, ^19^ and is being actively investigated in combination clinical studies in the treatment of relapsed or refractory AML. ^225^Ac‐lintuzumab delivers the ^225^Ac payload, a high energy, short path length, alpha emitting radionuclide directly to CD33‐positive myeloid tumor cells creating lethal double‐strand breaks in DNA and leading to selective tumor cell killing.^17^ One hit of an alpha particle emitted by ^225^Ac radionuclide can potentially kill a tumor cell, and the short path length focuses its radiation energy on targeted tumor cells, limiting exposure and damage to adjacent normal tissue.^20^ While external beam radiation has been combined with venetoclax in preclinical models, there is the potential to damage normal tissue. Further, scheduling radiation treatment relative to venetoclax administration may also be a challenge. As a potent inducer of DNA damage in targeted tumor cells, it is anticipated that ^225^Ac‐lintuzumab may mediate effective down‐modulation of MCL‐1 leading to a sensitization of AML cells to venetoclax irrespective of inherent resistance to the BCL‐2 inhibitor. Thus, the combination of potent targeted alpha radioimmunotherapy (RIT) plus venetoclax may be an effective combination strategy in AML. Here we describe the results of our in vitro and in vivo studies which evaluated ^225^Ac‐lintuzumab plus venetoclax in combination in established AML tumor cell lines exhibiting varying sensitivity to venetoclax. Unlike other strategies to deplete MCL‐1 in combination with venetoclax, our results demonstrate that ^225^Ac‐lintuzumab‐targeted internal alpha radiation exerts a dual mechanism of action, effecting potent single‐agent tumor killing through DNA double‐strand breaks, and the reduction in antiapoptotic proteins such as MCL‐1, leading to re‐sensitization of tumor cells to venetoclax and potent antitumor activity in preclinical models.

## 2 MATERIALS AND METHODS

### 2.1 Antibodies, reagents, and cell lines

Humanized anti‐CD33 antibody, lintuzumab, was provided by Actinium Pharmaceuticals, Inc.^17^ Venetoclax was purchased from Selleckchem (Cat. No.: S8048). For animal studies, venetoclax was formulated in 60% Phosal 50 PG (Cat. No.: NC0130871, Lipoid, Thermo Fisher Scientific), 30% polyethylene glycol 400 (Cat. No.: 81172, Sigma‐Aldrich), and 10% ethanol. ^225^Ac in anhydrous nitrate form was procured from Oak Ridge National Laboratory, USA. Bifunctional chelating agent p‐SCN‐Bn‐DOTA (DOTA) was purchased from Macrocyclics (Lot No.: B20510021). AML cell lines MOLM‐13 (DSMZ No.: ACC 554) and OCI‐AML3 (DSMZ No.: ACC 582) were purchased from Deutsche Sammlung von Mikroorganismen und Zellkulturen (DSMZ) and U937 from the ATCC (Cat. No.: CRL‐1593.2). All cell lines were cultured in RPMI‐1640 (Cat no: SH30255.01, Thermo Fisher Scientific) supplemented with 10% fetal bovine serum (Sigma‐Aldrich) and 1% antibiotic/antimycotic (Cat. No.: 15–240–062, Thermo Fisher Scientific). Cells were kept at 37^0^C in a 5% CO_2_ incubator. CD33 expression by the AML cell lines was determined by flow cytometry. Cells were stained with PE‐conjugated anti‐human CD33 or isotype control mAbs (Clone No.: HIM3‐4, Cat. No.: 12–0339–42, BD PharMingen).

### 2.2 Lintuzumab antibody conjugation and radiolabeling with ^225^Ac

The lintuzumab was attached to the bifunctional chelating agent *p*‐SCN‐Bn‐DOTA (Macrocyclics) by modified methodology reported in.^21^ Five hundred microgram of lintuzumab was attached to the bifunctional DOTA employing a 50‐fold molar excess of the chelating agent over antibody and incubated at 37°C for 1.5 h in 0.15 M sodium carbonate buffer, pH 8.5 (Cat. No.: 7527–04, VWR). The conjugation was followed by the purification of lintuzumab‐DOTA via buffer exchange into 0.15 M ammonium acetate, pH 6.5 (Cat. No.: 11599, VWR) at 4°C. The number of DOTA molecules attached to the lintuzumab molecule in the final conjugate was measured by MALDI‐TOF (University of Alberta) and determined to be approximately 10:1. The preservation of DOTA‐lintuzumab immunoreactivity toward CD33 antigen was confirmed by ELISA.

Lintuzumab‐DOTA conjugate was radiolabeled with ^225^Ac by dissolving ^225^Ac nitrate in 0.01 M HCl followed by 60 min incubation at 37°C with the conjugate at 37 kBq/µg ratio. DTPA (0.05 M solution) was used to stop the radiolabeling reaction and radiolabeling yield was determined by performing instant thin layer chromatography (iTLC) on Silica Gel‐Glass paper strips (Cat. No.: SG10001, Agilent Technologies) immediately after radiolabeling and counting the radioactivity on the iTLC strips 24 h after. At that time, ^225^Ac is in equilibrium with its daughter isotopes. A 2470 Wizard2 Gamma counter (Perkin Elmer) calibrated for the ^225^Ac radioactive daughter ^213^Bi emission spectrum was utilized to count radioactivity of iTLC strips. The radiolabeled antibody is found at the point of application on the iTLC strips, while ^225^Ac in form of ^225^Ac‐DTPA travels with the solvent front. Radiolabeling yields were greater than 99% and no further purification was required. The stability of ^225^Ac‐lintuzumab (formally known as ^225^Ac‐HuM195) in vitro and in vivo was determined previously^22^ and showed no leakage of Ac from the product.

### 2.3 In vitro AML cell cytotoxicity assay

U937 and OCI‐AML3 cells (2 × 10^5^ cells/mL) were cultured with 0–10 µM doses of venetoclax, whereas MOLM‐13 cells (2 × 10^5^ cells/mL) were cultured with doses ranging from 0 to 0.4 µM. AML cells (2 × 10^5^ cells/mL) were treated with unlabeled lintuzumab (0.1 μg), 0.74 kBq (0.02 μg), 1.48 kBq (0.04 μg), 2.22 kBq (0.06 μg), and 3.7 kBq (0.1 μg) ^225^Ac‐lintuzumab for 1 h, washed three times with PBS, and cultured. For combination experiments, AML cell lines (2 × 10^5^ cells/mL) were treated with 1.48 kBq ^225^Ac‐lintuzumab for 1 h, washed, and then incubated with 0.05 µM (MOLM‐13) or 0.5 µM (U937 and OCI‐AML3) venetoclax. Cells were cultured for 72 h and then cell death was measured using the tetrazolium dye (2, 3)‐bis‐(2‐methoxy‐4‐nitro‐5‐sulfophenyl)‐(2H)‐tetrazolium‐5‐carboxanilide (XTT) assay (Cat. No.: 11465015001, Sigma) according to manufacturer's instruction. After 72 h treatment, cells were incubated in the presence of 50 μl XTT (1 mg/mL), and 4 μL menadione (1 mM) for another 3 h and the absorbance was read at 492 nm in microplate reader (Spectra Max 250, Molecular Devices). The XTT proliferation assay was selected for use, as it has been widely utilized in cancer drug development studies to determine their cytotoxic effects. ^23^, ^24^

### 2.4 Phospho‐H2A.X assay for detection of DNA double‐strand breaks

The presence of phosphorylated H2A.X was measured by flow cytometry according to the manufacturer's instructions (Cat. No.: 17–344, Upstate Cell Signaling, Millipore Sigma). Briefly, U937 and OCI‐AML3 cells were treated with media, venetoclax alone (0.5 µM), ^225^Ac‐ Lintuzumab (1.48 kBq), or combination with venetoclax. After 72 h treatment, cells (2 × 10^6^ cells/ml) were washed twice with PBS and then resuspended in fixation solution (provided in kit). Cells were incubated for 20 min on ice and then washed twice with PBS. Subsequently, cells were resuspended in permeabilization solution (provided in kit) and stained with 3.5 µL of FITC‐conjugated anti‐phospho‐H2A.X (Ser139) or isotype control mouse IgG (provided in kit). After brief incubation for 20 min on ice, the cells were washed and resuspended in FACS buffer (1X PBS containing 0.2% BSA and 0.03% sodium azide). Stained samples were analyzed with a CytoFLEX flow cytometer (Beckman Coulter, Mississauga, ON) and data were analyzed using FlowJo software (Version 10, Tree Star Inc.).

### 2.5 Quantitative Western Blot

OCI‐AML3 cells were treated with media, unlabeled Lintuzumab or ^225^Ac‐Lintuzumab (1.48 and 3.7 kBq). Cells were collected 72 h posttreatment and lysed in ice‐cold lysis buffer (Cat. No.: 9803, Cell Signaling Technology) containing protease inhibitor PMSF (Cat. No.: 8553S, Cell Signaling Technology). Total protein was extracted in sample buffer (Cell Signaling Technology), sonicated, and boiled at 95^°^C for 10 min. For western blot assay, equal amounts of protein (15 µg per lane) were loaded to 10% sodium dodecyl sulphate‐polyacrylamide gel. After gel electrophoresis, wet electrotransfer (100v for 1 h) was performed to nitrocellulose membrane, 0.45 µm (pore size) (Cat. No.:1620115, Bio Red). Furthermore, membranes were blocked in blocking buffer (5% nonfat milk in TBST: 0.1% Tween‐20 in TBS) for 1 h at room temperature. Membranes were incubated with primary antibodies (Cell Signaling Technology), that is, human MCL‐1 (1:1000 dilution, Cat. No. 4572S), BCL‐2 (1:1000 dilution, Cat. No. 2872S), BCL‐XL (1:1000 dilution, Cat. No. 2764S), and anti‐mouse α‐tubulin (1:1000 dilution, Cat. No. 3873S) in blocking buffer with gentle agitation overnight at 4^0^C. Membranes were washed three times for 5 min each with TBST and then secondary detection was performed using Licor IRDye antibodies produced in goat (IRDye 680‐labeled anti‐rabbit 1: 10000, Cat. No.: 926–68071 and IRDye 800‐labeled anti‐mouse 1:10000 Cat. No.: 926–32210) for 1 h at room temperature. The protein bands were visualized by the LI‐COR Odyssey Imaging System and quantified by Image Studio lite (Version 5.2.5, LI‐COR Biosciences).

### 2.6 In vivo efficacy studies

Animal studies were approved by the University of Saskatchewan's Animal Research Ethics Board and followed to the Canadian Council on Animal Care guidelines for humane animal use. Tumor xenografts were established in 6‐week‐old female SCID mice (Strain code: 236, CB17/Icr‐Prkdc^scid^/IcrIcoCrl, obtained from Charles River Laboratories, Saint‐Constant, QC, Canada) by subcutaneous injection of 2x10^6^ OCI‐AML3 or U937 cells into the right flank. Tumor growth was measured with electronic calipers every 3 days (volume = length × width^2^/2). When tumors reached an volume of ~200 mm^3^, tumor‐bearing mice were randomized into five treatment groups of five animals and were treated with on day 1: IP injection of 0.4 μg unlabeled lintuzumab; IP injection of 7.4 kBq ^225^Ac‐lintuzumab; venetoclax (200 mg/kg) by oral gavage; combination of venetoclax and 7.4 kBq ^225^Ac‐lintuzumab; or left untreated. Mice in venetoclax alone and in combination treatment groups continued to receive venetoclax via oral gavage once daily over a period of 20 days. Mice body weights and tumor volumes were recorded three times per week. The animals were humanely euthanized when they experienced excessive weight loss (≤20%), or any tumor reached 4000 mm^3^ volume or became necrotic. The study was terminated at 38 days when the difference in survival between the treatment and control groups became significant. For safety study, blood was collected from euthanized mice and analyzed for liver toxicity (aspartate aminotransferase; AST and alanine transaminase; ALT) and kidney toxicity (creatinine and blood urea nitrogen; BUN).

### 2.7 Statistical analysis

Power analysis for the in vivo studies was estimated using PASS version 11 (NCSS, Inc.) using simulations of different tumor volumes based on pilot data and conservative assumptions regarding the groups treated with the radiolabeled antibodies. All simulations showed power of at least 83% with only five animals per group because of the large differences between treated and untreated animals. Thus, five mice per group were utilized in the in vivo studies. GraphPad Prism 7 was used to analyze all the data (GraphPad Software, Inc.). Differences between the treated and untreated groups in vitro were assessed using nonparametric Kruskal‐Wallis test with Dunn's correction for multiple comparisons. Error bars represent ±standard deviation (SD). Kaplan‐Meier data were analyzed by log‐rank (Mantel–Cox) test.

## 3 RESULTS

### 3.1 Single‐agent cytotoxicity of venetoclax and ^225^Ac‐lintuzumab in AML cell lines

**FIGURE 1:**
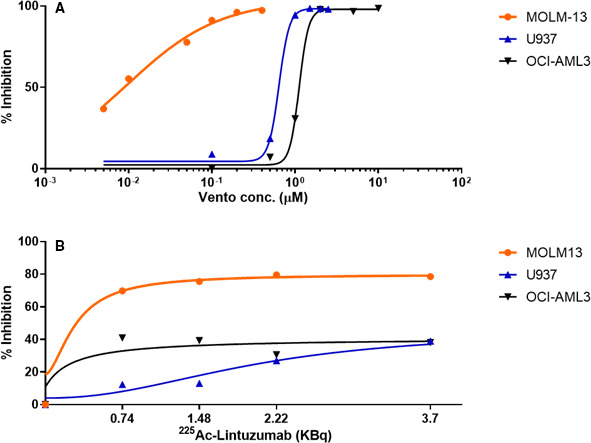
Single‐agent cellular cytotoxicity of venetoclax or ^225^Ac‐Lintuzumab treatment in AML cell lines. The AML cell lines (MOLM‐13, U937 and OCI‐AML3) were treated individually with increasing dose of venetoclax (A) or ^225^Ac‐Lintuzumab (B) and cell viability was measured using the XTT assay after 72 h posttreatment. For ^225^Ac‐Lintuzumab treatment, cells were treated with serial dilutions of ^225^Ac‐Lintuzumab for 1 h, washed, and then cultured for 72 h before performing the XTT assay. Data are shown as mean percentage of inhibition of three technical replicates per cell type. Experiment was repeated thrice with similar results.

Initially, two venetoclax resistant (OCI‐AML3 and U937) and one sensitive (MOLM‐13) AML cell lines were screened for CD33 expression. Flow cytometry analysis revealed high expression of CD33 in all three AML cell lines (Figure S1). To determine the optimal dose for in vitro combination studies, we evaluated the sensitivity of these cell lines to single‐agent venetoclax or ^225^Ac‐lintuzumab. The AML cell lines were treated individually with increasing concentrations of venetoclax or ^225^Ac‐lintuzumab and cell viability measured after 72 h by XTT assay. U937 and OCI‐AML3 cells began to show evidence of cytotoxicity at 0.5 µM venetoclax treatment, whereas MOLM‐13 cells were shown to be sensitive to concentrations as low as 0.005 µM (Figure 1A). The IC_50_ for venetoclax in MOLM‐13 was determined to be 0.0085 µM in comparison to venetoclax‐resistant lines U937 (IC_50 s_ 0.663 µM) and OCI‐AML3 (IC_50 s_ 1.115 µM). Treatment of U937 and OCI‐AML3 cells with ^225^Ac‐lintuzumab showed modest sensitivity following 1‐hour exposure to the antibody radio‐conjugate. MOLM‐13 was shown to be more sensitive to ^225^Ac‐lintuzumab at concentrations as low as 1.48 kBq (Figure 1B).

### 3.2 ^225^Ac‐lintuzumab and venetoclax combination shows synergetic cytotoxicity toward both sensitive and resistant AML cell lines

**FIGURE 2:**
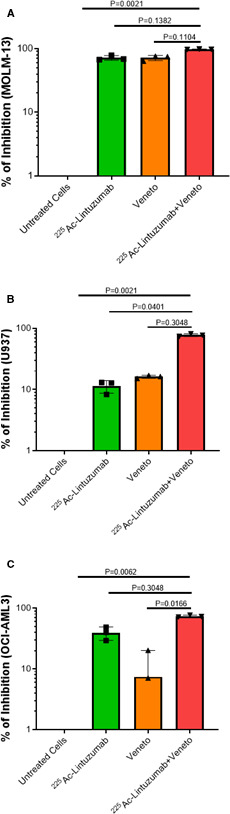
Combination treatment with ^225^Ac‐Lintuzumab and venetoclax (veneto) induces enhanced cytotoxicity in AML cell lines. MOLM‐13 (A), U937 (B) and OCI‐AML3 (C) cell lines were pre‐treated with ^225^Ac‐Lintuzumab for 1 h, washed, and then incubated with 0.05 µM (MOLM‐13) or 0.5 µM (U937 and OCI‐AML3) venetoclax for 72 h. Cell viability was measured by XTT assay. Data are shown as mean with SD of percentage of inhibition of three technical replicates per cell type. Experiment was repeated thrice with similar results. Statistics was calculated using nonparametric Kruskal‐Wallis test with Dunn's correction for multiple comparisons.

Based on results of single‐agent cytotoxicity of venetoclax and ^225^Ac‐lintuzumab, we performed studies to assess the potential for enhanced cell killing with the combination of ^225^Ac‐lintuzumab with venetoclax in both sensitive and resistant AML lines. In vitro combination cell cytotoxicity studies utilized IC_50_ concentrations determined from the single‐agent analyses: venetoclax, 0.05 µM (MOLM‐13) or 0.5 µM (U937 and OCI‐AML3) and 1.48 kBq (^225^Ac ‐lintuzumab). The cells were first exposed to ^225^Ac‐lintuzumab in the presence of venetoclax, then washed, and subsequently incubated in medium containing fresh venetoclax. Inhibition of cell growth was measured after 72 h posttreatment by XTT assay. Treatment with ^225^Ac‐lintuzumab combined with venetoclax resulted in greater tumor cell killing as compared to individual treatment in all three cell lines (Figure 2). Importantly, the enhancement of cell killing in the two venetoclax‐resistant lines—4.5‐fold increase in cell growth inhibition by combination treatment in comparison with single treatments for U937 cells and 2‐fold for OCI‐AML3 cells—suggests that the combination of venetoclax with ^225^Ac‐lintuzumab may affect re‐sensitization of these lines to venetoclax.

### 3.3 Initiation of double‐strand breaks in AML cells exposed to^225^Ac‐lintuzumab

**FIGURE 3:**
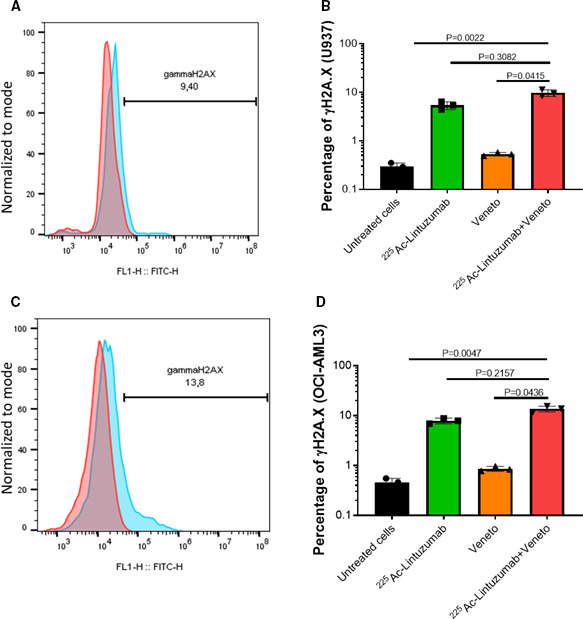
^225^Ac‐Lintuzumab induces double‐stranded DNA breaks in AML cell lines. U937 (A and B) and OCI‐AML3 (C and D) cells were treated with media, venetoclax alone (0.5 µM), ^225^Ac‐ Lintuzumab (1.48 kBq) or combination with venetoclax. The presence of phosphorylated gamma H2A.X was measured by flow cytometry after 72 h. On the histogram plots (A and C), red line shows binding of isotype control antibody and blue line shows binding of γH2A.X antibody. Data are shown as mean with SD of three technical replicates. Experiment was repeated twice with similar results. Statistics was calculated using nonparametric Kruskal‐Wallis test with Dunn's correction for multiple comparisons.

The ^225^Ac‐lintuzumab carries the ^225^Ac radionuclide generating potent high linear energy transfer alpha particles which can cause lethal DNA double‐strand breaks (DBSs). Upon induction of DNA damage, the nucleosomal histone protein H2A.X is rapidly phosphorylated at serine 139 to *γ*‐H2A.X at the DSBs site, making phosphorylated *γ*‐H2A.X a sensitive marker for detection of DNA DSBs.^25^ To define the potential mechanisms by which ^225^Ac‐lintuzumab induces significantly enhanced cellular cytotoxicity in AML cell lines, we screened for phosphorylated H2A.X following exposure to the antibody radio‐conjugate. Treatment of both U937 and OCI‐AML3 cells with^225^Ac‐lintuzumab alone or its combination with venetoclax induced high levels of *γ*‐H2A.X as compared to venetoclax with synergistic effect observed for combination treatment (Figure 3 and Figure S2).

### 3.4 ^225^Ac‐lintuzumab reduces MCL‐1, BCL‐2, and BCL‐XL levels in treated AML cells

**FIGURE 4:**
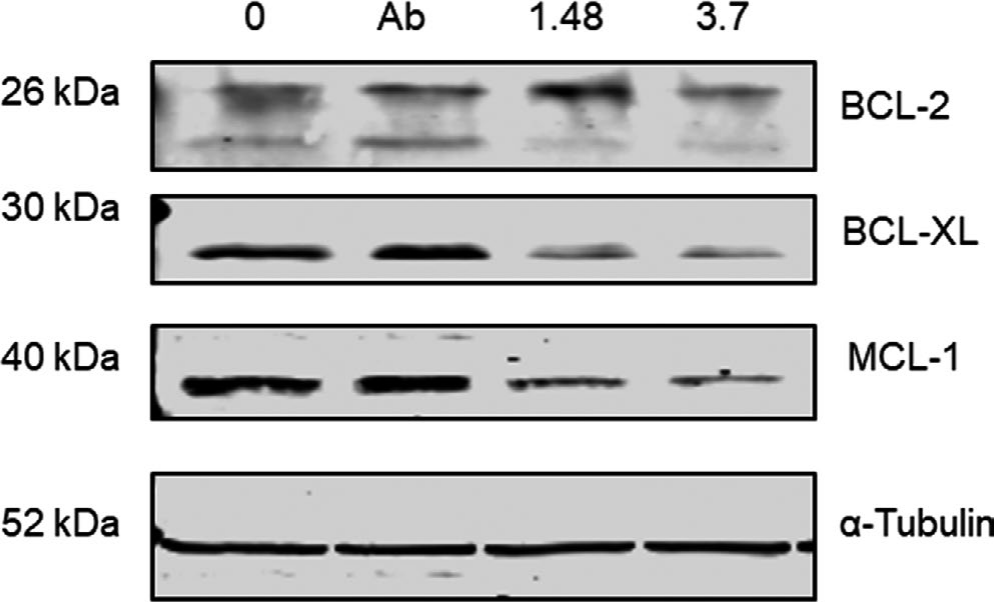
^225^Ac‐Lintuzumab downregulates the antiapoptotic MCL‐1, BCL‐2, and BCL‐XL protein levels in OCI‐AML3 cells. OCI‐AML3 cells were treated with media, unlabeled Lintuzumab or ^225^Ac‐Lintuzumab (1.48 and 3.7 kBq) for 1 h, washed, and further incubated for 72 h. Cells were collected 72 h posttreatment, lysed and Western blotting was performed. (A) LI‐COR Odyssey Imaging System was used to visualize the protein expression levels of MCL‐1, BCL‐2 and BCL‐XL proteins. Experiment was repeated twice with similar results.

It has been reported that genotoxic stress may inhibit the expression of BCL‐2 family proteins such as MCL‐1,^14^, ^15^ a protein implicated in resistance to BCL‐2 inhibitors such as venetoclax. To evaluate the potential for ^225^Ac‐lintuzumab to modulate MCL‐1 levels, we assessed the levels of antiapoptotic proteins in OCI‐AML3 cells via western blot. We determined that transient exposure of OCI‐AML3 cells to ^225^Ac‐lintuzumab significantly reduced the levels of not only MCL‐1, but also BCL‐XL, and to a lesser extent BCL‐2, in a dose‐dependent manner. This decrease in BCL‐2 family protein levels may serve as a mechanism by which this AML cell line can become re‐sensitized to venetoclax following combination treatment with ^225^Ac‐lintuzumab (Figure 4 and Figure S3).

### 3.5 Robust antitumor efficacy and survival of combination therapy in venetoclax‐resistant xenografts

**FIGURE 5:**
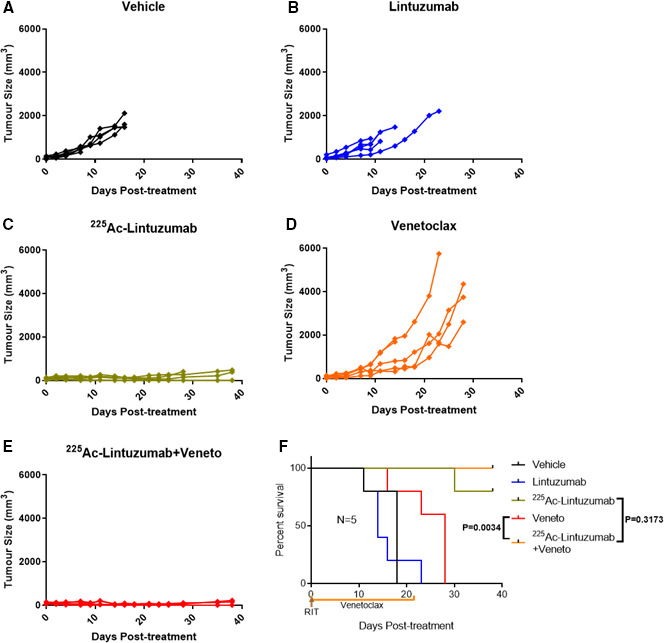
^225^Ac‐Lintuzumab and venetoclax combination effects a robust antitumor response and increases survival benefit in OCI‐AML3 xenografts. SCID mice (five animals per group) were injected with OCI‐AML3 cells into the right flank. Tumor‐bearing mice were treated with vehicle (A), unlabeled Lintuzumab (0.4 µg) (B), 7.4 kBq ^225^Ac‐Lintuzumab (0.2 µg) (C), venetoclax (200 mg/kg) (D), or combination of venetoclax and 7.4 kBq ^225^Ac‐Lintuzumab (E). Graph represents tumor volume of individual mice (N = 5). Tumor volume was calculated using the formula V = 0.5(LW^2^). (F) Kaplan‐Meier graph showing animal survival. Each curve represents five mice per group. P value was calculated using the log‐rank (Mantel‐Cox) test.

**FIGURE 6:**
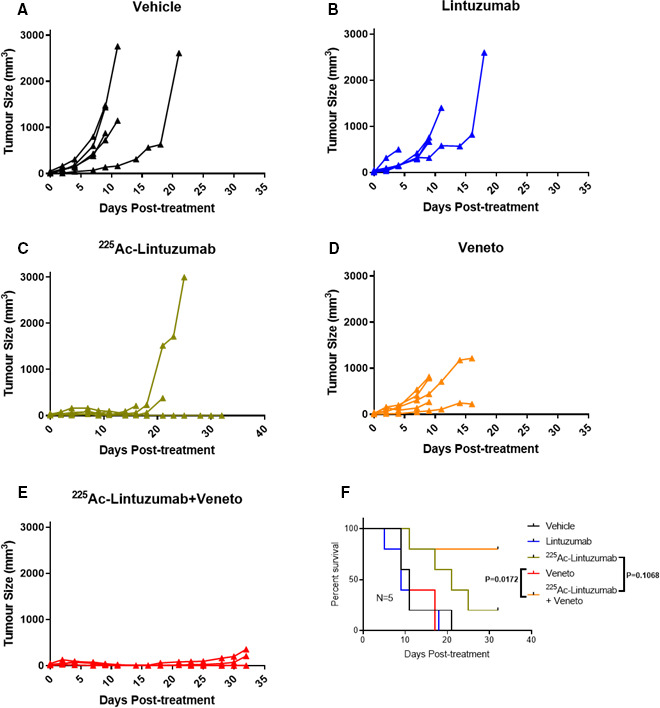
U937 xenografts model showed increased antitumor response and survival benefit with ^225^Ac‐Lintuzumab and venetoclax combination treatment. SCID mice (five animals per group) were injected with U937 cells into the right flank. Tumor‐bearing mice were treated with vehicle (A), unlabeled Lintuzumab (0.4 µg) (B), 7.4 kBq ^225^Ac‐Lintuzumab (0.2 µg) (C), venetoclax (200 mg/kg) (D), or combination of venetoclax and 7.4 kBq ^225^Ac‐Lintuzumab (E). Graph represents tumor volume of individual mice (N = 5). Tumor volume was calculated using the formula V = 0.5(LW^2^). (F) Kaplan‐Meier graph showing animal survival. Each curve represents five mice per group. P value was calculated using the log‐rank (Mantel‐Cox) test.

Since significant antitumor cell cytotoxicity was observed with combination treatment of ^225^Ac‐lintuzumab and venetoclax in AML cell lines, we evaluated this combination therapy in AML subcutaneous transplantation models using OCI‐AML3 and U937 xenografts in SCID mice. Vehicle‐treated, venetoclax‐treated, and single dose of naked unlabeled lintuzumab mAbs (0.4 µg) were unable to control tumor burden in mice in both models OCI‐AML3 (Figure 5A‐C) and U937 (Figure 6A‐C). Single‐agent venetoclax treatment showed no antitumor activity, although a modest survival benefit was observed in these resistant models compared to vehicle control and unlabeled lintuzumab antibody. Notably, both single‐agent ^225^Ac‐lintuzumab and ^225^Ac‐lintuzumab in combination with venetoclax significantly reduced tumor burden resulting in increased survival in both OCI‐AML3 (Figure 5D and E) and U937 (Figure 6D and E) models. Furthermore, at the end of the OCI‐AML3 animal study, on day 38 posttreatment, the group treated with 7.4 kBq ^225^Ac‐lintuzumab alone demonstrated 80% survival, with complete response in two of five mice (Table 1), while the group treated with the combination of ^225^Ac‐lintuzumab with venetoclax showed 100% survival and CR in three of five mice (Figure 5F). We chose to stop the study at 38 days' time point as by that time the difference in survival between the control and treatment groups became significant. Interestingly, in the U937 animal model, there was 80% survival with the combination of 7.4 kBq ^225^Ac‐lintuzumab with venetoclax and CR in two of five mice (Table 2), while the animals treated with the ^225^Ac‐lintuzumab alone demonstrated 20% survival (Figure 6F) with CR in one of five mice. Altogether these results demonstrate robust antitumor control and survival of the ^225^Ac‐lintuzumab combination with venetoclax in AML models refractory to single‐agent venetoclax.

**TABLE 1:** Viability^a^ and antitumor response^b^ to single agent and combination treatment in OCI‐AML3 model

Treatment	D21 Response	D38 Response
Vehicle	0/5 Alive 0/5 SD 0/5 PR 0/5 CR	0/5 Alive
Lintuzumab	1/5 Alive 0/5 SD 0/5 PR 0/5 CR	0/5 Alive
7.4 kBq^225^ Ac‐Lintuzumab	5/5 Alive 1/5 SD 2/5 PR 1/5 CR	4/5 Alive 0/5 SD 0/5 PR 2/5 CR
Venetoclax	4/5 Alive 0/5 SD 0/5 PR 0/5 CR	0/5 Alive
7.4 kBq^225^ Ac‐Lintuzumab + Venetoclax	SIS Alive 1/5 SD 2/5PR 2/5CR	5/5 Alive 0/5 SD 0/5 PR 3/5 CR

^a^ Mice were sacrificed if they experienced > 20% weight loss tumor volume reached 4000 mm^3^, or tumors became necrotic.

^b^ Anti‐tumor response was determined by change in tumor volume following treatment relative to tumor starting volume: SD, less than 30% change in tumor volume; PR. greater than 30% but less than 100% reduction in tumor volume; and CR. greater than 100% reduction in tumor volume.

**TABLE 2:** Viability^a^ and antitumor response^b^ to single agent and combination treatment in U937 model

Treatment	D21 Response	D32 Response
Vehicle	1/5 Alive 0/5 SD 0/5 PR 0/5 CR	0/5 Alive
Lintuzumab	0/5 Alive 0/5 SD 0/5 PR 0/5 CR	0/5 Alive
7.4 kBq^225^ Ac‐Lintuzumab	3/5 Alive 2/5 SD 0/5 PR 1/5 CR	1/S Alive 0/5 SD 0/5 PR 1/5 CR
Venetoclax	0/5 Alive 0/5 SD 0/5 PR 0/5 CR	0/5 Alive
7.4 kBq^225^ Ac‐Lintuzumab + Venetoclax	4/5 Alive 1/5 SD 1/5PR 2/5CR	4/5 Alive 0/5 SD 0/5 PR 2/5 CR

^a^ Mice were sacrificed if they experienced > 20% weight loss tumor volume reached 4000 mm^3^, or tumors became necrotic.

^b^ Anti‐tumor response was determined by change in tumor volume following treatment relative to tumor starting volume: SD, less than 30% change in tumor volume; PR. greater than 30% but less than 100% reduction in tumor volume; and CR. greater than 100% reduction in tumor volume.

### 3.6 Tolerable safety profile of ^225^Ac‐lintuzumab and venetoclax combination in tumor‐bearing mice

**FIGURE 7:**
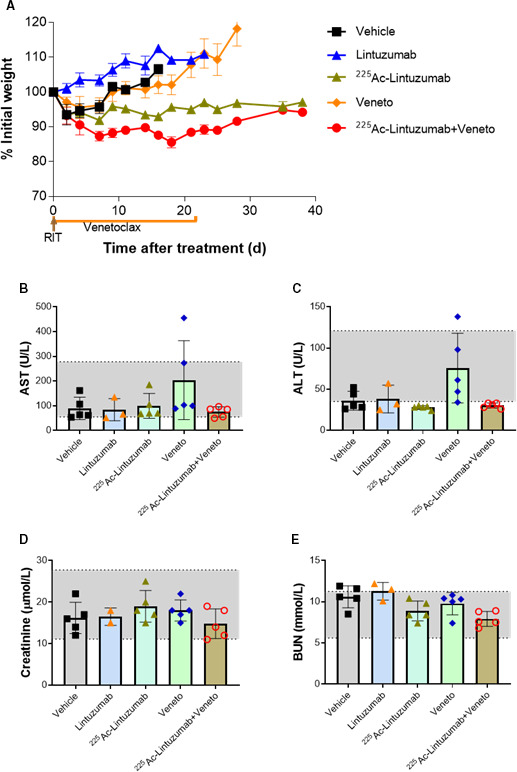
Combination treatment with ^225^Ac‐Lintuzumab and venetoclax showed tolerable safety profile in tumor‐bearing mice. OCI‐AML3 tumor‐bearing mice were treated as described in the legend for Figure 5. (A) Body weights were measured and recorded three times per week. Blood was collected from euthanized mice and analyzed for liver and kidney toxicity, that is, aspartate aminotransferase; AST (B), alanine transaminase; ALT (C), creatinine (D) and blood urea nitrogen; BUN (E). Data represent mean with SD of five animals per treatment group. The grey area represents normal ranges of the analytes for SCID mice.

A safety evaluation was performed on mice treated with venetoclax or ^225^Ac‐lintuzumab single agent or in combination by measuring the weight, liver toxicity (AST and ALT), and kidney toxicity (creatinine and BUN). ^225^Ac‐lintuzumab alone and the combination with venetoclax demonstrated transient but reversible weight loss (Figure 7A). There were no significant changes in AST (Figure 7B) and ALT (Figure 7C) levels in any of the treatment groups, though one of five mice in the venetoclax single‐agent cohort exhibited elevated enzyme levels. Furthermore, we did not find any evidence of increased creatinine (Figure 7D) and BUN (Figure 7E) levels indicating the absence of kidney toxicity in mice in any of the treatment groups.

## 4 DISCUSSION

Acute myeloid leukemia (AML) is a complex hematological disease often occurring in older patients. While several new targeted therapies have been recently approved, most patients eventually relapse and succumb to their disease. Strategic clinical approaches applying rational and scientifically supported therapeutic combinations are a logical step to further extend the response to, and benefit of, these novel targeted agents. To the best of our knowledge, this is the first study describing the combination of the recently approved venetoclax agent and radioimmunotherapy with an alpha emitter in AML. In this study, we demonstrated in vitro and in vivo that the combination of venetoclax with ^225^Ac‐lintuzumab, an antibody armed with the potent alpha emitting radionuclide Actinium‐225, had a synergistic effect with venetoclax on tumor cell killing in vitro and the combination provided a pronounced survival advantage in AML tumor models shown to be resistant and refractory to venetoclax.

BCL‐2 family proteins can promote tumor cell survival, making them attractive drug targets.^3^, ^26^, ^27^ Antagonists targeting BCL‐2/BCL‐XL (ABT‐737 and ABT‐263/navitoclax) or BCL‐2 only (ABT‐199/GDC‐0199/venetoclax) have shown clinical benefit, notably leading to the recent regulatory approval of venetoclax in CLL and AML. Not surprisingly, one mechanism of resistance to venetoclax is mediated by upregulation or overexpression of other BCL‐2 family members, including MCL‐1 and BCL‐XL.^7^, ^8^, ^9^, ^10^, ^11^ ^225^Ac‐lintuzumab targets the myeloid specific marker CD33 found overexpressed on most tumor cells in AML and MDS and less frequently in MM.^17^, ^28^, ^29^ Phase 1 and 2 studies have demonstrated evidence of single‐agent clinical activity, supporting further investigation in combination with other therapies in the treatment of myeloid malignances.^18^, ^19^ Importantly, ^225^Ac‐lintuzumab internalizes on binding to its CD33 antigen into the cells and effectively carries ^225^Ac inside the cells^22^ thereby limiting off‐target toxicities and contributing to a high degree of patient tolerability in clinical trials.^17^, ^18^, ^19^ In the present study, we demonstrated robust antitumor activity and survival from the combination of ^225^Ac‐lintuzumab with venetoclax in AML lines refractory to the BCL‐2 inhibitor. It has been shown that DNA damage can reduce the expression of MCL‐1 protein.^15^, ^30^ Therefore, the use of targeted therapies that increase tumor‐specific DNA damage may be a promising approach to enhance the potency of venetoclax and overcome resistance mechanisms. As anticipated, exposure of AML cell lines to ^225^Ac‐lintuzumab potently induced DNA double‐strand breaks and demonstrated single‐agent activity in vitro and in vivo. Importantly, the combination with venetoclax had an apparent synergistic effect on DNA damage that in turn reduced the expression of MCL‐1 protein leading to a significant enhancement of antitumor potency in AML cell lines resistant to single‐agent venetoclax. To investigate the possible mechanisms by which ^225^Ac‐lintuzumab may potentiate or re‐sensitize resistant AML lines to the venetoclax, we assessed the impact of the antibody radio‐conjugate on cellular levels of antiapoptotic proteins MCL‐1, BCL‐2, and BCL‐XL. Previously, Niu et al.^31^ have screened 11 AML cell lines and showed wide range of venetoclax sensitivity. Based on that study, we have selected two highly venetoclax resistant (OCI‐AML3 and U937) and one sensitive (MOLM‐13) AML cell lines to cover the wide range of sensitivities to single‐agent venetoclax—the differences between IC50 for the sensitive MOLM‐13 and resistant OCI‐AML3 and U937 were 131‐ and 78‐fold, respectively. Interestingly, OCI‐AML3 cells exposed to ^225^Ac‐lintuzumab demonstrated significantly reduced levels of both MCL‐1 and BCL‐XL compared to controls. Mechanistically, the reduction of both MCL‐1 and BCL‐XL was likely the result of genotoxic stress induced in tumor cells because of alpha particle‐mediated DNA damage. The reduction of BCL‐2 family proteins by ^225^Ac‐lintuzumab effectively mitigates resistance to venetoclax in AML tumor lines. There might be additional mechanisms involved in the combination treatment such as radiosensitizing effect of venetoclax. In this regard, O'Steen et al., who combined radioimmunotherapy with beta‐emitter ^90^Y and venetoclax, and observed the synergistic results of such combination, refer to “multiple mechanisms” of action.^16^ The radiosensitizing nature of venetoclax warrants future studies.

As a result, the combination induced high DNA damage which in turn caused a pronounced antitumor response and survival benefit in vivo in mice bearing venetoclax‐resistant AML xenografts. In these models, a single dose of ^225^Ac‐lintuzumab was enough to induce a durable response compared to a 21‐day course of venetoclax. The combination therapy also had very favorable toxicity profile including absence of kidney toxicity. In this regard, no kidney toxicity was observed in clinical trials of ^225^Ac‐lintuzumab.^17^, ^18^ We anticipate that successive courses of such combination therapy would enable a sustained response, extending survival with curative intent. At present, there is significant interest in the clinical evaluation of venetoclax and drug combinations with direct or indirect inhibitors of MCL‐1 to overcome resistance to the BCL‐2 inhibitor.^32^ Mechanistically, the combination of ^225^Ac‐lintuzumab antibody radio‐conjugate therapy with venetoclax offers the dual benefit of not only mediating single‐agent antitumor activity, but also, as demonstrated in this study, effects the reduction of both MCL‐1 and BCL‐XL. By reducing MCL‐1 and BCL‐XL, ^225^Ac‐lintuzumab reverses resistance to venetoclax. As such, this therapeutic combination may exhibit superior response in AML in comparison with combinations solely focused on suppression of BCL‐2 pathways.

Other groups have been investigating the utility of different radionuclides targeting CD33 antigen or different antigens/radionuclides as potential targets and cytotoxic moieties for radioimmunotherapy of AML in experimental models. In this regard, Hagemann et al. targeted CD33 on leukemic cells with an antibody armed with a long‐lived alpha emitter ^227^Thorium.^33^ Though this proof of principle in vivo study was somewhat limited in scope with only one HL‐60 xenograft used, the results were encouraging in terms of efficacy and toxicity. O'Steen et al. utilized pretargeted RIT (PRIT) system directed against the CD20 antigen on leukemic cells and observed tumor reduction with a beta‐emitter ^90^Y.^16^ Very recently, the same group investigated targeting CD45 antigen on leukemic cells with the novel bispecific fusion proteins targeting CD45 and ^90^Y‐DOTA ^34^ and observed tumor reduction, confirming the potential of beta emitters in radioimmunotherapy of AML. Bergstrom et al. used a different approach by targeting interleukin‐3 receptor α‐subchain (CD123), which is overexpressed relative to the β‐subchain (CD131) on leukemia stem cells with an Auger‐emitter ^111^In‐labeled DTPA‐NLS‐CSL360 radioimmunoconjugate.^35^ In contrast to the beta‐emitter, the use of an Auger‐emitter resulted in paradoxical radiation priming effect of radioimmunotherapy on increasing the hCD45(+) cell population in the bone marrow and spleen of NOD/SCID or NRG mice.

Overall, our results support the clinical testing of the ^225^Ac‐lintuzumab in combination with venetoclax in the treatment of venetoclax‐resistant AML. Clinical trial of this combination therapy (NCT03867682) is currently ongoing.

## CONFLICT OF INTEREST

The research was funded by Actinium Pharmaceuticals and the funder had input into designing of the study and writing the manuscript. ED received the funding from Actinium Pharmaceuticals. DL and EG are employees of Actinium Pharmaceuticals. The rest of the authors declare no conflict of interest.

## Supporting information

Fig S1‐S4

## Data Availability

The data that support the findings of this study are available from the corresponding author upon reasonable request.
