# Supplementary material for: 225Ac‐labeled CD33‐targeting antibody reverses resistance to Bcl‐2 inhibitor venetoclax in acute myeloid leukemia models
Source: Cancer Med. 2020 Dec 21;10(3):1128–40. doi: 10.1002/cam4.3665 (PMC7897952; doi:10.1002/cam4.3665)
Supplement: Supplementary file 1 — Fig S1‐S4 [file CAM4-10-1128-s001.pptx]

## Slide 1
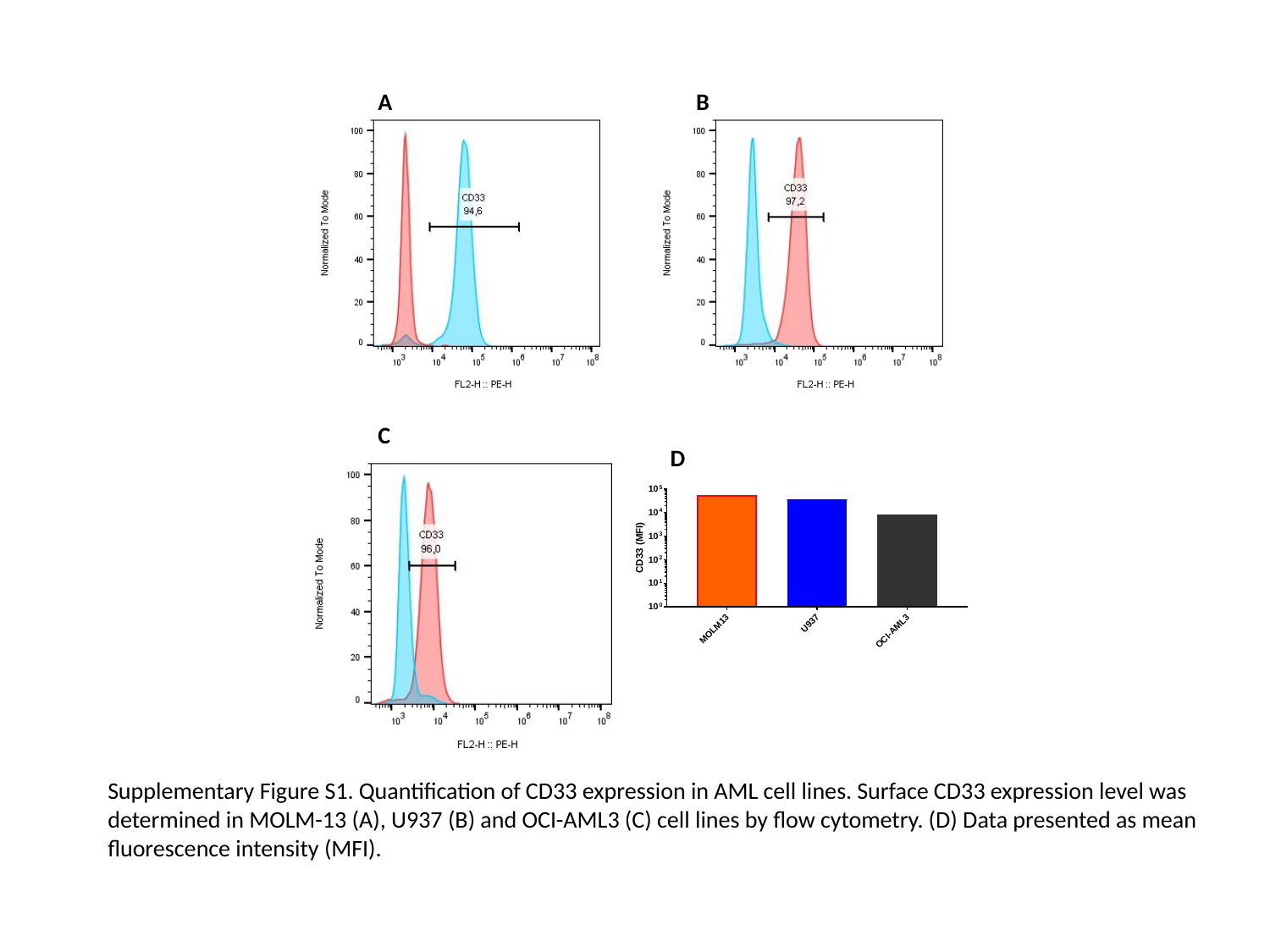

A
B
C
D
Supplementary Figure S1. Quantification of CD33 expression in AML cell lines. Surface CD33 expression level was determined in MOLM-13 (A), U937 (B) and OCI-AML3 (C) cell lines by flow cytometry. (D) Data presented as mean fluorescence intensity (MFI).

## Slide 2
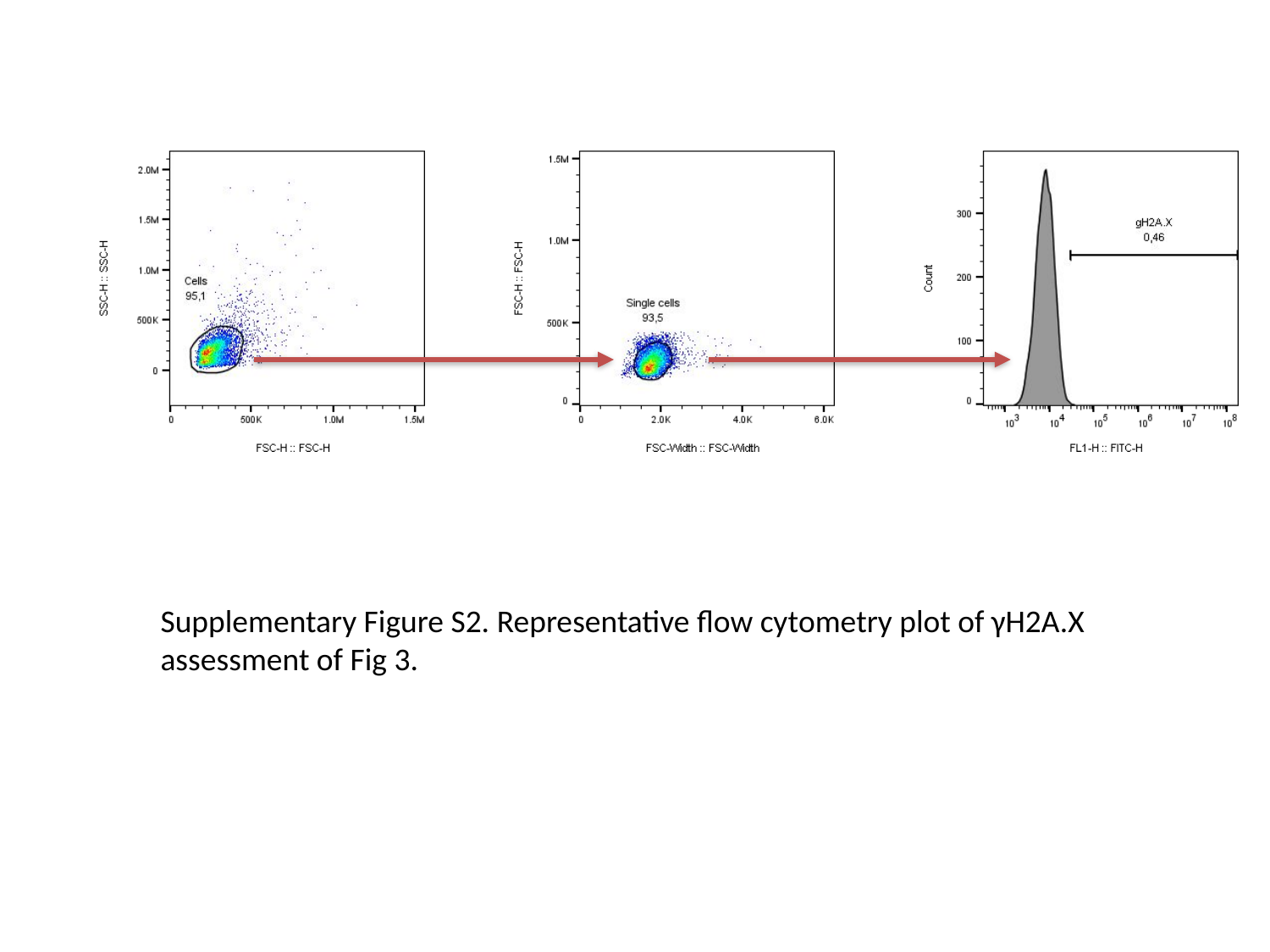

Supplementary Figure S2. Representative flow cytometry plot of γH2A.X assessment of Fig 3.

## Slide 3
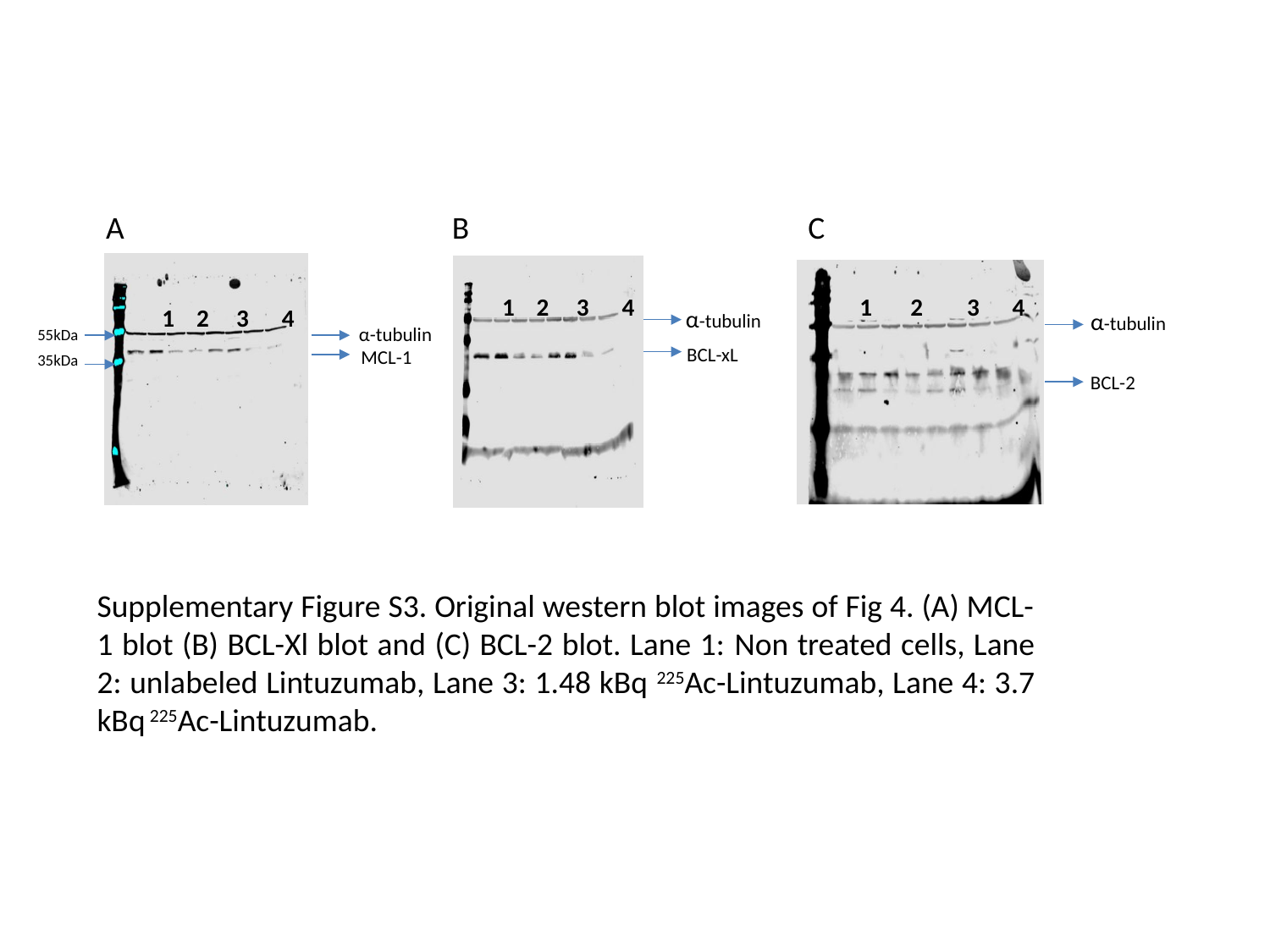

A
B
C
1 2 3 4
1 2 3 4
1 2 3 4
α-tubulin
α-tubulin
α-tubulin
BCL-xL
MCL-1
BCL-2
55kDa
35kDa
Supplementary Figure S3. Original western blot images of Fig 4. (A) MCL-1 blot (B) BCL-Xl blot and (C) BCL-2 blot. Lane 1: Non treated cells, Lane 2: unlabeled Lintuzumab, Lane 3: 1.48 kBq 225Ac-Lintuzumab, Lane 4: 3.7 kBq 225Ac-Lintuzumab.

## Slide 4
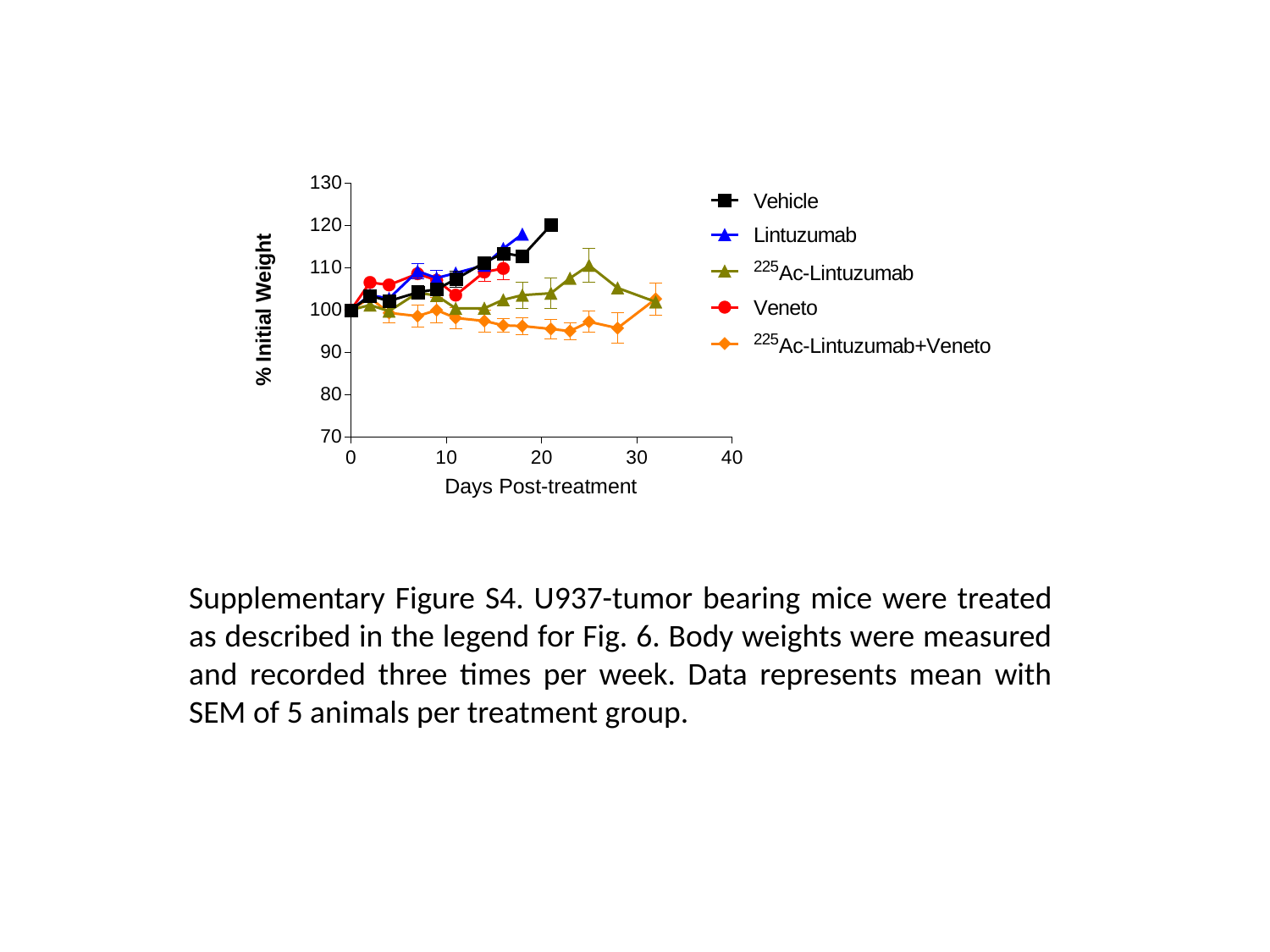

Supplementary Figure S4. U937-tumor bearing mice were treated as described in the legend for Fig. 6. Body weights were measured and recorded three times per week. Data represents mean with SEM of 5 animals per treatment group.
